# Thy1-YFP: an effective tool for single cell tracing from neuronal progenitors to mature functionally active neurons

**DOI:** 10.1038/s41420-025-02297-z

**Published:** 2025-01-22

**Authors:** Ante Plećaš, Katarina Kapuralin, Leonarda Grandverger, Dinko Mitrečić, Ivan Alić

**Affiliations:** 1 Department of Anatomy, Histology and Embryology, Faculty of Veterinary Medicine, University of Zagreb, Zagreb, Croatia; 2 Faculty of Biotechnology and Drug Development, University of Rijeka, Rijeka, Croatia; 3 Croatian Institute for Brain Research, School of Medicine, University of Zagreb, Zagreb, Croatia

**Keywords:** Cellular neuroscience, Neural stem cells

## Abstract

The differentiation of mouse neurons is a complex process involving cell maturation and branching, occurring during both, embryonic development and differentiation in vitro. To study mouse neuronal morphology, we used the Thy1 YFP-16 mouse strain. Although this mouse strain was described over twenty years ago, detailed studies on projections outgrowth and morphology of neurons are still lacking. The main goal of our study was to analyse the differentiation patterns of neural stem cells, including markers of differentiation, colocalization patterns, synaptic markers and the tracing of cell projections during differentiation in vitro. The neural stem cells were isolated from embryos at embryonic day 14.5 as well as newborn pups and differentiated into neurons and astrocytes. Our data showed a significant decrease of neural stem cells markers and a substantial increase in neuronal markers during differentiation, analysed by immunocytochemistry, quantitative PCR and western blot. To assess synaptic maturation, neurons were further analysed by quantitative PCR and immunocytochemistry. Expression of synaptic markers were increased during differentiation in vitro. At the 7^th^ day in vitro differentiation, expression of synaptic markers in both YFP positive and YFP negative neurons were at comparable levels. Finally, our data revealed a significant increase in all measured morphological parameters: Filament Area, Filament Length, Filament No. Terminal Points and Sholl Intersections in YFP positive/MAP2 positive neurons compared to YFP negative/MAP2 positive neurons. These findings suggest that YFP is an effective tool for cell tracing both in vivo and in vitro, making it valuable for morphological studies during development as well as in the context of neurodegenerative disorders.

## Introduction

During embryonic development neuronal progenitors are organised into a 3D structure and follow a unique pattern of differentiation [1]. This process, which includes cell maturation and branching, is also observed during differentiation in vitro. Although routine in vitro cell cultures lack the 3^rd^ dimension, 2D neuronal differentiation offers several benefits, including: (a) individual cell tracing, (b) analysis of synaptic activity and (c) analysis of cell branching. Detailed analyses of growth of neuronal projections during differentiation improves our understanding of not only development of mammalian brain tissue, but also mechanisms underlying the onset and progression of pathological events and regeneration. However, even in such a simplified in vitro system, the thickness and number of neuronal projections make the analyses of neuronal branching highly complex. While mouse neuronal morphology has been well described, detailed studies on neuronal branching are still limited. The main goal of our study was to analyse the pattern of neural stem cells (NSCs) differentiation, focusing on markers of differentiation, Pearson’s coefficient of colocalization, synaptic markers, outgrowth of cell projections, and cell death during differentiation in vitro. To perform a detailed analyses of the number, length and shape of neuronal projections over time, we used a transgenic mouse strain, that expresses neuron-specific yellow fluorescent protein (YFP) [2] under the control of *Thy1* gene in motor axons, retina, dorsal root ganglia, cervical ganglia, cortex (2–6 layer) and cerebellum. In our previous work, the same strain was used for NSCs transplantation into mouse brains affected by stroke [3–6]. Moreover, recent studies have made use of different Thy1-YFP mouse strains to study dendritic spine morphology [7–11], axonal morphology and pathology [12–15], neuromuscular junctions [16], tumours and wound-healing [17], retinal morphology and pathology [18, 19], the cochlea [20] and the trigeminal ganglion [21]. A key advantage of this particular mouse strain is the relatively small proportion of YFP positive cells, approximately 20% [3] of the total neuronal population, which allows for clear visualization of the entire cell, including its projections.

One of the main questions in neuronal morphological studies is whether neurons remain functionally active during differentiation in vitro. Although development and functional maturation of synapses have been well studied in the developing mammalian central nervous system (CNS) in vivo [22, 23] and comparison has been made between cortical synapses in primates and mice [24, 25] using various animal models [26], therefore, we aimed to determine whether Thy1-YFP neurons express synaptic markers and at what day in vitro (DIV) they reach a plateau of expression.

By using NSCs from both E14.5 and P0, we revealed that in vitro differentiation mirrors the events that occur during in vivo development. Moreover, we observed the presence of GFAP positive astrocytes in mature neuronal culture, while Thy1-YFP colocalised with neuronal markers in vitro. Our findings suggest that YFP is a valuable tool for cell tracing both in vivo and in vitro, making it an effective resource for morphological studies during development as well as in the investigation of neurodegenerative disorders.

## Results

### The differentiation of NSCs in vitro mirrors the events that occur during development in vivo

**Fig. 1:**
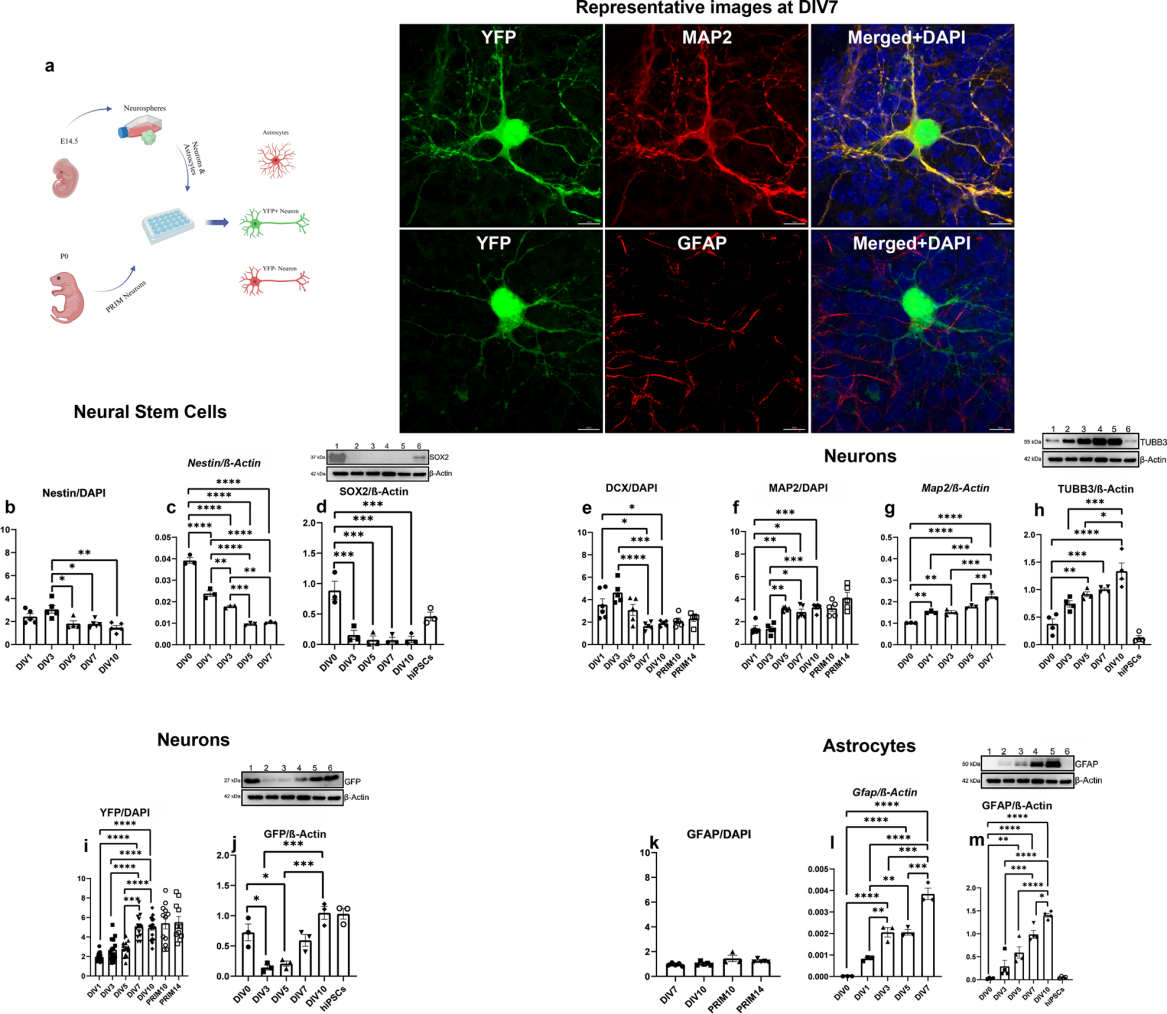
Analyses of Thy1-YFP cells during differentiation in vitro. The schematic images of experiments were created using BioRender.com and representative confocal images at DIV7. Scale bar 10 µm (**a**). Neural Stem Cells: During differentiation in vitro, significant decrease of Nestin expression was obtained by ICC (**b**) and qPCR (**c**) while the significant decrease of SOX2 was obtained by WB (**d**). Neurons: significant decrease of DCX (immature neurons) was obtained by ICC (**e**), significant increase of MAP2 (mature neurons) were obtained by ICC (**f**) and qPCR (**g**), significant increase of TUBB3 was obtained by WB (**h**), significant increase of YFP was obtained by ICC (**i**) while the significant increase of GFP was obtained by WB (**j**). Astrocytes: Astrocytes were observed in mature culture by ICC (**k**) while the significant increase was obtained by qPCR (**l**) and WB (**m**). Human iPSCs were used as a control sample (**d**, **h**, **j**, **m**). *P* values are provided in the separate Supplementary document 1. Graphs represent means ± SEM.

For the purpose of this study, we analysed both neurons obtained by differentiation of NSCs and primary (PRIM) neurons in vitro (Fig. 1a). To compare the pattern of differentiation in vitro with the same process of normal embryonic development, we analysed the expression of Nestin, MAP2 and Thy1-YFP during embryonic development and postnatal age. As neuronal progenitors reach its peak at E13.5, the optimal ratio between Nestin positive cells and Thy1-YFP positive progenitors was observed at E14.5 (Supplementary Fig. 1a, b), and between MAP2 and Thy1-YFP in the P0 cortex and hippocampus (Supplementary Fig. 1c). Based on these results, NSCs were isolated from the telencephalic wall of E14.5 and P0. Additionally, in order to analyse cell proliferation and ratio of living and death cells, BrdU labelling and LIVE/DEAD kit were used. These analyses revealed that more than 75% of NSCs were BrdU positive (Supplementary Fig. 2), and 100% of NSCs were live at DIV0 (Supplementary Fig. 3). Finally, to confirm the neuron specific Thy1-YFP expression, NSCs were differentiated into astrocytes, and no YFP positive astrocytes were detected at DIV10 (Supplementary Fig. 4).

During differentiation in vitro Nestin (neuroepithelial stem cell protein) expression showed a significant, gradual decrease in neurons obtained from NSCs differentiation. No difference was observed between DIV1 and DIV3, while significant differences were observed between DIV3 and DIV5 (*p* = 0.0256), as well as between DIV3 and DIV7 (*p* = 0.0135). Finally, a twofold decrease in Nestin expression was seen between DIV3 and DIV10 (*p* = 0.0016) (Fig. 1b, Supplementary Fig. 5a). To validate these findings obtained by immunocytochemistry (ICC), qPCR was performed (Fig. 1c). The highest *Nestin* expression was obtained in neurospheres (DIV0) with a significant decline (*p* < 0.0001) at each subsequent time point during the seven-day differentiation period. Significant differences were noted between the following time points: DIV1 and DIV3 (*p* = 0.0069), DIV1 and DIV5 (*p* < 0.0001), DIV1 and DIV7 (*p* < 0.0001), DIV3 and DIV5 (*p* = 0.0006), and DIV3 and DIV7 (*p* = 0.0013).

In order to show NSCs differentiation at the WB level, SRY homology box (SOX2) antibody was used (Fig. 1d). The highest SOX2 (transcription factor involved in maintenance of embryonic and neural stem cells) expression was observed in neurospheres (DIV0), with a threefold decrease compared to DIV3 (*p* = 0.0008), and similarly low levels at subsequent time points (DIV5-DIV10, *p* = 0.0003). Human iPSCs were used as a positive control for SOX2 expression. Finally, we quantified the percentage of Paired box protein 6 (PAX6, additional transcription factor expressed in ectodermal cells)-positive cells during differentiation in vitro. The highest percentage (12%) of PAX6 positive cells was observed at DIV1 with a onefold decrease compared to the later time points (Supplementary Fig. 6a). These results demonstrate that NSCs differentiation in vitro closely mirrors developmental processes observed in vivo, with a notable decrease in NSCs markers Nestin and SOX2 over time.

### DCX is expressed at the same time as Nestin during differentiation in vitro

Doublecortin (DCX), a well-known marker of immature neurons, was highly expressed at DIV1 and reached its peak at DIV3, comparable to Nestin as NSCs media was gradually changed to the neuronal differentiation media. Significant differences were observed between DIV1 and DIV7 (*p* = 0.0136), DIV1 and DIV10 (*p* = 0.0498), DIV3 and DIV7 (*p* < 0.0001), and between DIV3 and DIV10 (*p* = 0.0003). This indicates that DCX is expressed in neuronal precursors alongside Nestin. During the first five days of differentiation in vitro, the DCX/Nestin ratio was 1.5, but after day 5, it dropped to 1.0, suggesting these markers are co-expressed in the same cells. Moreover, DCX expression in PRIM neurons was consistent with levels observed in mature neurons obtained by differentiation of NSCs at DIV7 and DIV10 (Fig. 1e, Supplementary Fig. 5b). Additionally, the percentage of T-box transcription factor (TBR2) positive cells, another immature neuronal marker, was quantified. During the first five days of differentiation in vitro, approximately 4% of TBR2 positive cells were observed, with a two-fold decrease at DIV7 and DIV10. The same percentage was observed in PRIM neurons (Supplementary Fig. 6b). These results indicate that the expression of TBR2 is downregulated as neuronal differentiation progresses, suggesting a transition from immature to more mature neuronal states.

### Mature neurons reach a plateau of differentiation after DIV7 in vitro

To analyse neuronal maturation, the following neuronal markers were used: MAP2 (a pan-neuronal marker expressed in the perikaryon and dendrites), TUBB3 (a pan-neuronal marker), CTIP2 (nuclear marker), SATB2 (nuclear marker) and SMI-312 (axonal marker).

Although MAP2 (Microtubule Associated Protein 2) was scarcely expressed under our conditions at DIV1, the maximum increase occurred between DIV5 and DIV10, when its peak expression was observed. Significant differences were obtained between DIV1 and DIV5 (*p* = 0.0022), DIV7 (*p* = 0.0163), and DIV10 (*p* = 0.0009), as well as between DIV3 and DIV5 (*p* = 0.0027), DIV7 (*p* = 0.00124) and DIV10 (*p* = 0.0007). Our data showed a twofold increase in MAP2 expression during differentiation in vitro, suggesting full neuronal maturation. Additionally, MAP2 expression in PRIM neurons was at the same level as in mature neurons obtained by differentiation of NSCs at DIV7 and DIV10, with no significant difference between PRIM10 and PRM14, further supporting evidence of complete maturation and differentiation (Fig. 1a, f, Supplementary Fig. 5c). Similar results were obtained at mRNA level (Fig. 1g). The lowest *Map2* expression was detected in neurospheres (DIV0), with a significant increase during seven days of differentiation in vitro: DIV1 (*p* = 0.0028), DIV3 (*p* = 0.0028), DIV5 (*p* < 0.0001), and DIV7 (*p* < 0.0001). Moreover, significant differences were observed between: DIV1 and DIV7 (*p* < 0.0001), DIV3 and DIV7 (*p* < 0.0001), and DIV5 and DIV7 (*p* = 0.0034).

To evaluate pan-neuronal expression at WB level Tubulin Beta 3 (TUBB3) antibody was used. The lowest TUBB3 expression was detected in neurospheres (DIV0) with a two- to threefold increase during differentiation in vitro: DIV5 (*p* = 0.0021), DIV7 (0.0004) and DIV10 (*p* < 0.0001). Significant differences were observed between DIV3 and DIV10 (*p* = 0.0007), and between DIV5 and DIV10 (*p* = 0.0222). Human iPSCs were used as a negative control for TUBB3 expression (Fig. 1h).

Additionally, to quantify the percentage of cortical neurons, two nuclear markers were used: CTIP2 (transcription factor interacting protein 2) and SATB2 (Special AT-rich sequence-binding protein 2). The highest percentage of CTIP2 positive cells (12%) was observed at DIV1, with a slight decrease during differentiation in both, neurons obtained by NSCs differentiation and PRIM neurons (Supplementary Fig. 6c). Conversely, the highest percentage of SATB2 positive cells (6%) was observed at DIV5. Similar level of SATB2 expression was noted in PRIM neurons as well (Supplementary Fig. 6d). These results indicate that, although in small proportion, deep layer cortical neurons (CTIP2, layer V) and upper layer cortical neurons (SATB2, layer II/III) were present during differentiation in vitro and mirrors the events that occur during mouse cortical development.

To analyse axons in both mature neurons obtained by NSCs differentiation and in PRIM neurons, SMI-312 (Cocktail, composed of three axon-specific neurofilaments) antibody was used. The SMI-312 expression was analysed in mature neurons only, as earlier time points were SMI-312 negative; it remained constant and there were no significant differences between time points or different protocols of differentiation (Supplementary Fig. 7). That result suggests that axonal differentiation and outgrowth in PRIM neurons follow the same pattern.

Finaly, we analysed expression of neuron-specific YFP signal during differentiation in vitro. YFP showed significant differences between: DIV1 and DIV7 (*p* < 0.0001), DIV1 and DIV10 (*p* < 0.0001), DIV3 and DIV7 (*p* < 0.0001), DIV3 and DIV10 (*p* < 0.0001), and finally between DIV5 and DIV7 (*p* = 0.0001), and DIV5 and DIV10 (*p* < 0.0001). No differences were observed between earlier time points or between the two PRIM neuron time points. Moreover, YFP expression in PRIM neurons was at the same level as in mature neurons obtained by differentiation of NSCs (Fig. 1i). However, since YFP is a spectral variant of GFP, GFP expression was also analysed by WB. The highest GFP expression was observed in neurospheres (DIV0), with significant differences compared to DIV3 (*p* = 0.0123) and DIV5 (*p* = 0.0293). Significant differences were also noted between DIV3 and DIV10 (*p* = 0.0002), and between DIV5 and DIV10 (*p* = 0.0005). Human GFP labelled iPSCs were used as a positive control for GFP expression (Fig. 1j). The highest expression at DIV10 may be due to increased cell volume (signal), likely a result of cell maturation. Altogether, our data indicate that neurons achieve full maturation after DIV7 in vitro, with consistent expression of key neuronal markers across both NSCs-derived and PRIM neurons, confirming the reliability of these models of differentiation for studying neuronal development.

### GFAP positive astrocytes were present in mature neuronal culture

In addition to neuron-specific markers, the astrocyte-specific marker GFAP was also analysed by ICC, qPCR and WB. To assess astrocyte expression, cells were stained with a GFAP antibody, reviling positive astrocytes in both, cells cultures obtained by differentiation of NSCs at DIV7 and DIV10, as well as in PRIM cells at DIV10 and DIV14 (Fig. 1a, k). Although the cells were directed towards neuronal differentiation, a small percentage of GFAP positive astrocytes, which were YFP negative, were still observed under our conditions. The qPCR analysis showed that *Gfap* expression was absent in neurospheres (DIV0), but a significant increase in expression was observed over time, with significant differences noted at all time points (*p* < 0.0001). Significant differences were obtained between DIV1 and DIV3 (*p* = 0.0038), DIV1 and DIV5 (*p* = 0.0038), DIV1 and DIV7 (*p* < 0.0001), DIV3 and DIV7 (0.0002), and between DIV5 and DIV7 (*p* = 0.0002) (Fig. 1l). Similar results were obtained at protein level: no expression was detected in neurospheres (DIV0). Significant differences were found between DIV0 and DIV5 (*p* = 0.0022), DIV7 (*p* < 0.0001) and DIV10 (*p* < 0.0001), between DIV3 and DIV7 (*p* = 0.0002), DIV3 and DIV10 (*p* < 0.0001), DIV5 and DIV10 (*p* < 0.0001) and between DIV7 and DIV10 (*p* = 0.0354). Human iPSCs were used as a negative control for GFAP expression (Fig. 1m). These findings indicate that a small but significant population of GFAP positive/YFP negative astrocytes were present in the cultures, with GFAP expression increasing significantly over time.

### Thy1-YFP colocalise with neuronal markers in vitro

**Fig. 2:**
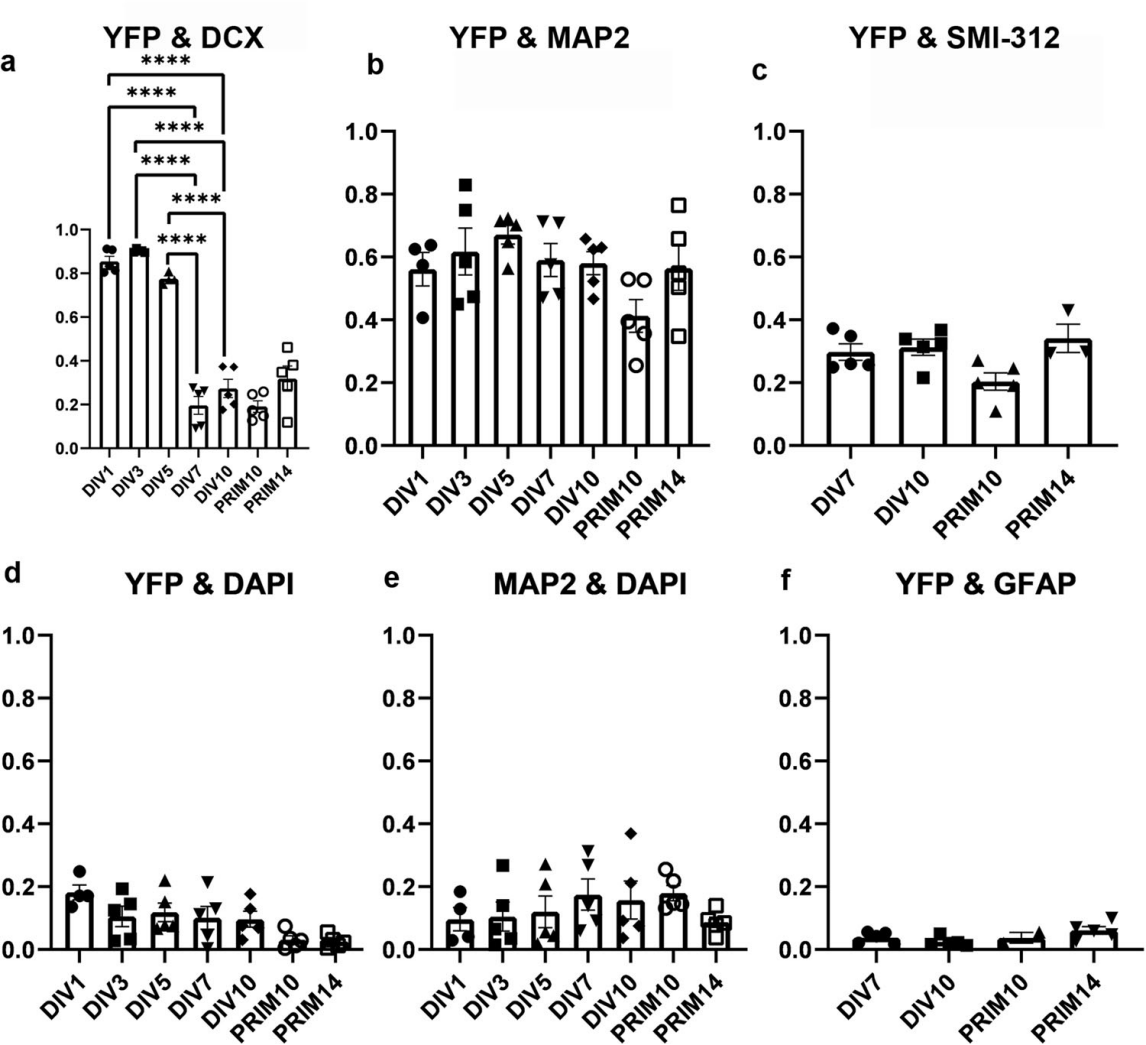
Analysis of Pearson’s coefficient of colocalization during differentiation in vitro. A high Pearson’s coefficient of colocalization was observed between YFP and DCX during the first five days of differentiation in vitro. After 5 days, in both mature neurons obtained by differentiation of NSCs and PRIM neurons, Pearson’s coefficient of colocalization was low, at background level (**a**). A high and consistent Pearson’s coefficient of colocalization was observed between YFP and MAP2 during differentiation in vitro (**b**). A weak Pearson’s coefficient of colocalization was shown between YFP and SMI-312 in mature neuros (**c**). As a control, Pearson’s coefficient of colocalization was calculated between YFP and DAPI, MAP2 and DAPI, and between YFP and GFAP to show background levels of colocalization (**d**–**f**). *P* values are provided in the separate Supplementary document 1. Graphs represent means ± SEM.

To quantify the extent of YFP colocalization with specific cell populations, Pearson’s coefficient of colocalization between YFP and various neuronal and astrocyte markers was calculated using IMARIS software. A high Pearson’s coefficient of colocalization (0.8) between YFP and DCX was obtained during the first five days of differentiation. However, in mature neurons at DIV7 and DIV10, as well as in PRIM neurons, the Pearson’s coefficient of colocalization dropped to background level (0.2). This decrease was expected, as mature neurons do not express DCX, while YFP positive cells were fully mature and branched (Fig. 2a). The Pearson’s coefficient of colocalization between YFP and MAP2 was 0.6 in both neurons obtained by differentiation of NSCs and PRIM neurons (Fig. 2b). These findings demonstrated similar pattern of MAP2 and YFP expression during differentiation in vitro shown in Fig. 1f, i. In mature neurons Pearson’s coefficient of colocalization between YFP and SMI-312 was 0.3 in both, neurons obtained by differentiation of NSCs and PRIM neurons (Fig. 2c). As a control, Pearson’s coefficients were calculated between YFP and DAPI (0.02) (Fig. 2d), MAP2 and DAPI (0.02) (Fig. 2e) as well as between YFP and GFAP (0.02) (Fig. 2f), which all remained at background levels and show no colocalization. This data further supports our finding that neurons achieve full maturation after DIV5 in vitro, as evidenced by the high colocalization of YFP with early neuronal markers during initial differentiation and its reduction to background levels in mature neurons and astrocytes, confirming YFP’s neuronal specificity.

### Presence of YFP does not affect synaptic expression in mature neurons

**Fig. 3:**
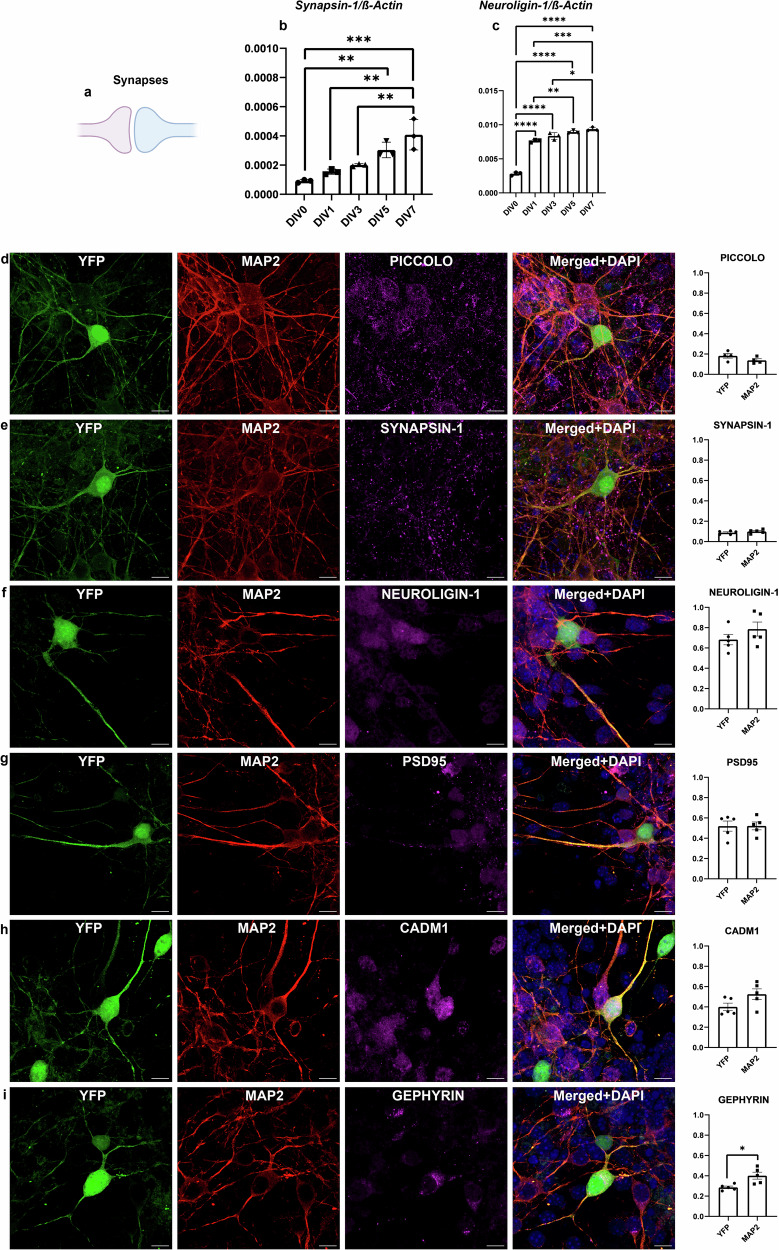
Analysis of synaptic markers. The schematic image of synapses was created using BioRender.com (**a**). qPCR analysis of the *Synapsin-1* (**b**) and *Neuroligin-1* (**c**) during differentiation in vitro, with significant differences between time points. Fluorescent analysis of synaptic markers in mature neurons at DIV7. The expression of synaptic markers was consistent in both YFP positive and YFP negative/MAP2 positive masked neurons, as indicated by the presynaptic markers PICOLLO (**d**) and SYNAPSIN-1 (**e**), and postsynaptic markers NEUROLIGIN-1 (**f**), PSD95 (**g**) and CADM1 (**h**), with the exception of GEPHYRIN (**i**), which showed a small but significant difference. Scale bar 10 µm. *P* values are provided in the separate Supplementary document 1. Graphs represent means ± SEM.

In order to analyse maturation and functional activity of Thy1-YFP cells during differentiation in vitro, mature neurons obtained by differentiation of NSCs at DIV7 were stained with multiple synaptic markers. Specifically, two presynaptic markers (PICCOLO and SYNAPISN-1) and four postsynaptic markers (NEUROLIGIN-1, PSD95, CADM1 and GEPHYRIN) were used (Fig. 3). We chose DIV7 as the representative time point for analysis for several reasons: (a) the neurons were morphologically mature and fully branched, (b) our qPCR data showed the highest expression of *Synapsin-1* with significant differences between: DIV0 and DIV5 (*p* = 0.0063), DIV0 and DIV7 (*p* = 0.0003), DIV1 and DIV7 (*p* = 0.0017), between DIV3 and DIV7 (*p* = 0.0076) (Fig. 3b) and *Neurologin-1* with significant differences between DIV0 and the following time points: DIV1, DIV3, DIV5 and DIV7 (*p* < 0.0001), and between DIV1 and DIV5 (*p* = 0,0042), DIV1 and DIV7 (*p* = 0.0004) and between DIV3 and DIV7 (*p* = 0.0239) (Fig. 3c), and (c) we aimed to compare YFP positive neurons with YFP negative neurons. To achieve this, YFP positive neurons were masked using IMARIS software, and synaptic vesicles were analysed within the masked cells. MAP2-masked cells were used as the second group for comparison. This approach revealed that synaptic expression was comparable between YFP positive/MAP2 positive and YFP negative/MAP2 positive masked neurons. Therefore, no significant differences in synaptic marker expression were observed between these two cell populations. GEPHYRIN was the only marker that showed a small but significant difference between YFP and MAP2 (*p* = 0.0135) (Fig. 3i). These results led us to further investigate neuronal branching during 10 days of differentiation in vitro, as well as in PRIM neurons at DIV10 and DIV14.

### Expression of YFP in Thy1-YFP is sufficient for neuronal tracing

**Fig. 4:**
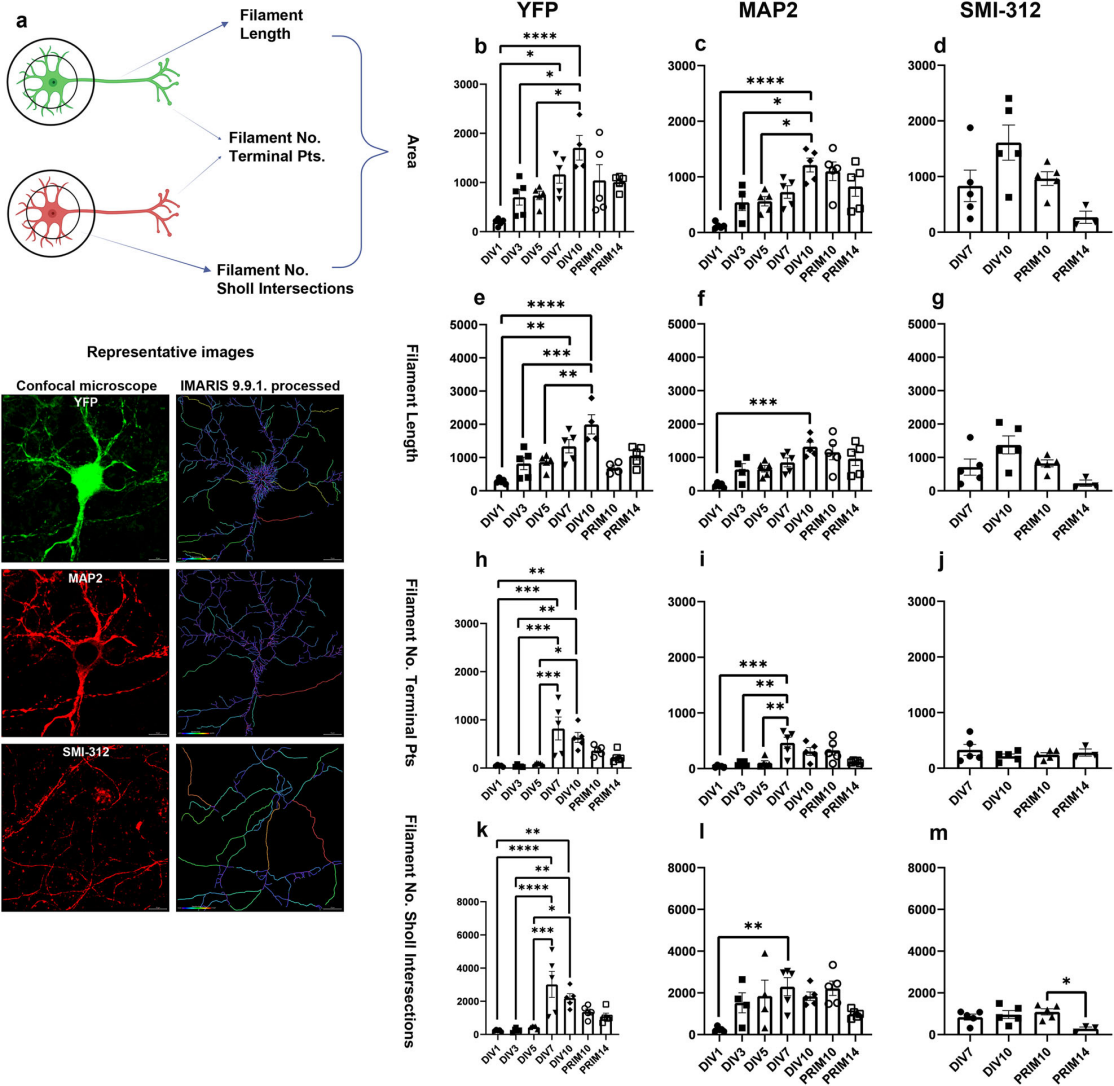
Filament analysis of YFP positive/MAP2 positive and YFP negative/MAP2 positive cells as well as SMI-312 positive axons during differentiation in vitro. The schematic images of experiments were created using BioRender.com, representative confocal images and the corresponding IMARIS-processed images at DIV7. Scale bar 10 µm (**a**). Significant difference in total Filament Area, Filament Length, Filament No. Terminal Pts and Filament No. Sholl Intersection was observed between YFP and MAP2 neurons obtained by differentiation of NSCs as they matured. However, no significant differences were found between the two time points in PRIM neurons (**b**, **c**, **e**, **f**, **h**, **i**, **k**, **l**). SMI-312 expression was consistent in mature neurons differentiated from NSCs and PRIM neurons (**d**, **g**, **j**), with a small, but significant difference in the Filament No. Sholl Intersection between the two time points in PRIM neurons (**m**). *P* values are provided in the separate Supplementary document 1. Graphs represent means ± SEM.

To investigate neuronal branching, we analysed Filaments (cell projections) using the Neuroscience mode of IMARIS software. We traced individual YFP-masked cell, MAP2-masked cells and SMI-312-labelled axons. Design of experiment, representative confocal images of analysed cells and their corresponding IMARIS-processed images are shown in Fig. 4a. We measured the total Filament Area (Fig. 4b–d), Filament Length (Fig. 4e–g), Filament Number of Terminal Points (Fig. 4h–j) and Filament Number of Sholl Intersections (Fig. 4k–m).

When analysing the YFP Filament Area per field of view, the smallest Filament Area was observed at DIV1, with a significant increase at DIV7 (*p* = 0.0119) and DIV10 (*p* < 0.0001). Significant differences were also noted between DIV3 and DIV10 (*p* = 0.0155) and between DIV5 and DIV10 (*p* = 0.022). Notably, neurons obtained by differentiation of NSCs covered a larger Area per field of view compared to PRIM neurons, likely due to differences in cell density, differentiation, and synaptic maturation. PRIM cells, being more challenging to manipulate, resulted in fewer mature neurons per field of view. No significant differences were found between two time points for PRIM neurons (Fig. 4b). Similarly, YFP Filament Length showed significant differences between: DIV1 and DIV7 (*p* = 0.0013), DIV1 and DIV10 (*p* < 0.0001), DIV3 and DIV10 (*p* = 0.0006), and DIV5 and DIV10 (*p* = 0.001) (Fig. 4e). Further analysis of the number of Filament Terminal Points revealed highly significant differences between the following time points: DIV1 and DIV7 (*p* = 0.0002), DIV1 and DIV10 (*p* = 0.0071), DIV3 and DIV7 (*p* = 0.0002), DIV3 and DIV10 (*p* = 0.0062), DIV5 and DIV7 (*p* = 0.0005), and finally DIV5 and DIV10 (*p* = 0.0157) (Fig. 4h). The Number of Filament Sholl Intersections per field of view showed similarly significant differences between: DIV1 and DIV7 (*p* < 0.0001), DIV1 and DIV10 (*p* = 0.0049), DIV3 and DIV7 (*p* < 0.0001), DIV3 and DIV10 (*p* = 0.0058), DIV5 and DIV7 (*p* = 0.0001), and DIV5 and DIV10 (*p* = 0.0161) (Fig. 4k).

A similar analysis was performed on MAP2 positive cells per field of view. In MAP2 positive neurons, significant differences in the total Area were observed between DIV1 and DIV10 (*p* < 0.0001), DIV3 and DIV10 (*p* = 0.00352), and between DIV5 and DIV10 (*p* = 0.0267) (Fig. 4c). MAP2 Filament Length also showed a significant difference between DIV1 and DIV10 (*p* = 0.0003) (Fig. 4f). The Number of MAP2 positive Filament Terminal Points differed significantly between DIV1 and DIV7 (*p* = 0.0004), DIV3 and DIV7 (*p* = 0.002), and between DIV5 and DIV7 (*p* = 0.0039) (Fig. 4i). Finally, the number of Filament Sholl Intersections per field of view showed a significant difference between DIV1 and DIV7 (*p* = 0.0093) (Fig. 4l).

Additionally, SMI-312-labelled axons were analysed in mature neurons only, as earlier time points were SMI-312 negative. SMI-312 expression levels were comparable between neurons obtained by differentiation of NSCs and PRIM neurons (Fig. 4d, g, j). A small, but significant difference in the number of Filament Sholl Intersections were observed between PRIM10 and PRIM14 (*p* = 0.0340) (Fig. 4m).

When comparing Filament tracing between YFP positive/MAP2 positive and YFP negative/MAP2 positive neurons, a clear difference in significance was immediately evident, with higher statistical significance in YFP compared to MAP2 positive cells. Several factors may account for this: (a) the culture was confluent, (b) only 20% of the neurons were YFP positive, (c) YFP is expressed in all cell compartments, (d) MAP2 is expressed primarily in the perikaryon and dendrites, and (e) using a 60x objective, we captured a single YFP positive cell with numerous MAP2 positive cells per field of view. It was easier to trace a single YFP positive cell with all its projections compared to the confluent MAP2 positive neurons. To assess synaptic activity (Fig. 3), the cells were grown in a confluent culture. A compelling result supporting our hypothesis is shown in Fig. 4, where the number of Filament Sholl Intersections in YFP positive cells showed a highly significant difference in mature, fully differentiated, and synaptically active cells (DIV7-10) compared to the earlier time points. Based on these results, we conclude that YFP is a highly effective tool for studying neuronal morphology and cell tracing both in vitro, as well as in vivo.

## Discussion

To create a comprehensive picture of the complex processes involved in neuronal differentiation, it is essential to integrate data from both in vitro and in vivo experiments collected using different methodologies, and techniques. In neuroscience, various approaches have been used to trace and/or label specific cells or cell types. For example, Lipofectamine transfection [27] has been used, although it typically yields low efficiency in post-mitotic neurons (around 1–5%). In our experience, this efficiency can reach approximately 10%, depending on the cell type. Another approach involves the micro-injection of dyes such as Lucifer Yellow [28] and Alexa Fluor Hydrazide [29] to visualize dendritic spines and localization of synapses. Transgenic animal models are also widely used, including mice that express GFP in all cells [30], those that conditionally express GFP via the Cre-Lox System [31], or transgenic mice that express YFP [2] in approximately 20% neurons [3] under the control of *Thy1* gene. The greatest advantage of the Thy1-YFP mouse strain is that YFP is neuron-specific, expressed in all neuronal compartments, and is expressed in a relatively small subset of neurons. Moreover, unlike transfection or cell injection methods, there is no need for aggressive cell manipulation.

While dendritic spines have been extensively studied and well documented in the literature [7–11], we focused on the morphology of cellular processes during differentiation in vitro. The main goal of our study was to analyse the pattern of NSCs differentiation, including markers of differentiation, Pearson’s coefficient of colocalization, synaptic markers, as well as to trace the outgrowth of cell projections during differentiation in vitro using the Thy1-YFP mouse strain. First, we demonstrated robust YFP expression during embryonic development (Supplementary Fig. 1). Based on our extensive experience, the optimal ratio of YFP positive/Nestin positive NSCs was obtained from E14.5 embryos, which were then used for differentiation. To ensure quality of the cells, we performed BrdU labelling, which revealed over 75% positive cells after 8 h of incubation (Supplementary Fig. 2), and confirmed that 100% NSCs were alive at DIV0, with fewer than 40% of cells dying by DIV10, as differentiation and maturation progressed (Supplementary Fig. 3). To validate and confirm neuron-specific expression of YFP [2], NSCs were differentiated into astrocytes. Although we started with neurospheres containing approximately 20% YFP positive cells, the neuronal progenitors, including YFP positive cells, died during astrocytic differentiation, while the glia-progenitors differentiated into mature astrocytes (Supplementary Fig. 4).

In order to analyse the morphology of neuronal processes during differentiation in vitro, we examined the expression of synaptic markers at DIV7 to show neuronal maturation using qPCR and ICC (Fig. 3), and previously published protein level data [5]. To the best of our knowledge, no detailed morphological analysis of Thy1-YFP neurons and its processes in vitro has been conducted so far. Using the Neuroscience mode of IMARIS 9.9.1. software we observed a significant increase of Filament Area per field of view in both YFP positive and MAP2 positive neurons. This reflects the extension of neuronal processes and the formation of a complex network with strong synaptic connections during differentiation. Similarly, Filament Length was increased in MAP2 positive neurons and also dramatically increased in YFP positive neurons during differentiation. These results can be attributed to the relatively small proportion of Thy1-YFP positive neurons, approximately 20%, as we previously reported [3]. Using an Olympus 60x objective, we captured and analysed individual YFP positive/MAP2 positive cell alongside multiple YFP negative/MAP2 positive cells per field of view. By acquiring full Z-stacks, we were able to image YFP positive cells in 3D and analyse their full length starting from the nucleus. This approach was applied to each neuron in the field, with YFP positive neurons providing a clear signal and distinct boundary compared to MAP2 positive neurons. We observed significant differences in the Filament No. Sholl Intersections of YFP positive cells during differentiation, indicating how many times their processes of YFP positive cells intersected concentric circles centred on the nucleus. While YFP negative cells also exhibited significant differences, tracing them was more challenging, and the significance was reduced in YFP negative cells using the same parameters. All analysed parameters reached their highest values in mature, synaptically active neurons, as shown in Fig. 3 and our previous work [5]. Furthermore, to validate our findings, all experiments were also performed on PRIM neurons derived from P0, which yielded similar results to neurons obtained by differentiation of neurospheres. Although, YFP is expressed in all processes, axons were labelled with the pan-axonal marker (SMI-312) in mature neurons, where no significant differences were observed between time points. However, an increasing trend was noted. This may be due to imaging limitations, as the 60x objective has a relatively small frame, making it difficult to analyse every single axon and trace it back to its origin.

A similar approach has been employed by other groups to study glia cells, focusing on their connections with dendritic spines and their role in hippocampal excitatory synapses [32], microglia activation after penetrating traumatic brain injury [33] and the structural remodelling of the central amygdala, microglia and astrocytes during heart failure that affected cell volume, surface area, filament length, and glial branching [34].

In addition to IMARIS software, other machine-learning-based tools for image analysis, such as ilastik [35] and LABKIT [36], have been used. Recently, our group also published a new tool, Lusca, which is designed for analysis of neurons, organelles, and blood vessels [37, 38].

In conclusion, using the Neuroscience mode of IMARIS software, we provided a detailed morphological analysis of processes in Thy1 neurons in both YFP positive/MAP2 positive and YFP negative/MAP2 positive cells. The main findings of this research suggest that YFP is a highly effective tool for cell tracing both in vivo and in vitro, making it valuable resource for morphological studies during development and in the context of neurodegenerative disorders.

## Materials and methods

### Experimental animals

Experiments were carried out on B6.Cg-Tg(Thy1-YFP)16Jrs(Thy1 YFP-16) mouse strain on C57B1/6NCrl background which expresses *Thy1*-driven yellow fluorescent protein (YFP), expressed in the neurons. The animals were kept in Animal Facility of the Croatian Institute for Brain Research at the temperature 22 +/− 2 °C with 55% +/−10% humidity and 12/12 h light/night circle with access to pelleted food and water *ad libitum*. All animal procedures were approved by Internal Review Bord of the Ethical Committee of the School of Medicine University of Zagreb (04-77/2010-238) as well as Faculty of Veterinary Medicine (251/61-01/139-13-4) and in accordance with European Union Directives 2010/63/EU on the protection of animals used for scientific purposes.

### Neural stem cells isolations and cultivation

After sacrifice of Thy1 YFP-16 pregnant females (n = 3), NSCs were isolated from telencephalic wall of E14.5 (n = 6/litter). Developing brain tissue was first dissected and minced with scissors in Phosphate Buffered Saline solution (PBS, Gibco by Life Technologies, 14190-144) and after that, dissociation of single-cell suspension was obtained using Accutase (Gibco by Life Technologies, A11105-01) at 37 °C for 20 min. The cell suspension was centrifuged at 300 × *g* for 5 min, supernatant was removed and isolated cells were placed in sterile flasks (BD falcon, 353110) together with proliferation supporting medium consisting of DMEM/F-12 (Gibco by Life Technologies, 31331-028) supplemented with antibiotics Penicillin Streptomycin (Gibco by Life Technologies, 15140-122), B-27 Supplement (Gibco by Life Technologies, 17504-044), N2 Supplement (Gibco by Life Technologies, 17502-048), EGF (Recombinant Mouse Epidermal Growth Factor, PMG8041) and FGFb (Recombinant Mouse Fibroblast Growth Factor, PMG0035). After two days neuorspheres were formed and for the purpose of differentiation analyses were dissociated by Accutase and plated on 12 mm coverslips density of 200–250,000 cells per coverslip and 6-well plates density of 1 × 10^6^ cells per well. Coverslips and 6 well plates were previously coated with Geltrex (Gibco by Life Technologies A14133-02). Cells were plated in a medium without growth factors and were placed in incubator for 24 h. After 24 h medium was changed into Neurobasal (Gibco by Life Technologies, 21103-0499) in addition of antibiotic with glutamine (Pen Strep Glutamine, Gibco by Life Technologies, 10370-016). Cells were fixed with 4% PFA on the following Days In Vitro differentiation (DIV): 1, 3, 5, 7 and 10. Additionally, some coverslips were differentiated to the astrocytes using DMEM/F-12 supplemented with antibiotics and 10% Fetal Bovine Serum (Gibco by Life Technologies, 10270-106).

Except for the in vitro differentiation, three E14.5 embryos were PFA fixed, cryoprotected, sliced and stained to show YFP expression during embryonic development.

Primary (PRIM) neurons were obtained from the telencephalic wall of newborn pups (P0) (n = 3 pups/litter, in total 9 pups). Tissue was processed as we describe above, seeded on coated coverslips immediately after isolation in the same media as neurons obtained by differentiation of NSCs. PRIM neurons were fixed at DIV 10 and 14.

### Immunostaining

Cells were fixed with 4% PFA for 15 min. After washing with PBS cells were ready for immunostaining (immunohistochemistry (IHC) & immunocytochemistry (ICC)). Immunostaining was performed as previously described [3]. Shortly, before immunolabeling, cells were blocked with blocking solution [0.2% triton X-100 (Sigma, T8787-100ML) in PBS + 3% goat serum] at room temperature (RT) for 60 minutes. After 1 h, cells were rinsed 3 × 5 min in PBS and labelled with primary antibodies at 4 °C O/N (Supplementary Table 1). Next day cells were rinsed 3 × 5 min in PBS, after which secondary antibodies were added and incubate at RT for next 2 h (Supplementary Table 2). Secondary antibodies were rinsed with PBS and nuclei were counter-stained with DAPI (Sigma, D9542). Embryonic tissues were stained following the same protocol.

### BrdU labelling

NSCs were labelled with 10 μM 5-Bromo-20-deoxyuridine (BrdU, Sigma, B5002-1G,) for 8 h. Labelled cells were seeded on previously coated coverslips as it described above, and ICC was performed.

### LIVE/DEAD cell imaging kit

Discrimination between live and dead cells was performed using Live/Dead Cell Imaging kit (Invitrogen, R37601). Briefly, two probes, live (green vial) and dead (red vial) were mixed to obtain 2x stock solution. Cells at the DIV0 and DIV10 were incubated with stock solution at RT for 15 min. Fluorescent figures were captured using EVOS FL Auto Imaging System.

### Western blot

Cells for WB were harvested at the following DIV: 0, 3, 5, 7 and 10, and stored at −80 °C. Cells were lysed in RIPA buffer with addition of phosphatase and protease inhibitors. Bradford Assay Reagent (ThermoFisher SCIENTIFIC, 1863028) was used for quantification of protein concentrations. Samples were run on a 12% stain-free gel (BIO-RAD, 1610185) and after electrophoretic separation transferred to a nitrocellulose membrane (Millipore, IPVH00005) according to the manufacturers protocols (Bio-Rad). Membranes were blocked using a 5% low fat milk (Sigma-Aldrich, 70166-500G) in TBST at RT for 1 h and incubated O/N with primary antibodies (Supplementary Table 1). Afterwards, the membranes were rinsed 3 times in TBST and incubated with appropriate secondary antibodies at RT for 1 h (Supplementary Table 2). The bands were visualized with SuperSignal™ West Femto Maximum Sensitivity Substrate (ThermoFisher SCIENTIFIC, 34095) while quantification was carried out using BioRad software and density was calculated by Fiji-win 64 software using “gel analysis” option. SOX2, TUBB3, GFP and GFAP signal was normalised with β-Actin. Full length blots are provided in the Supplementary information - Original data.

### qPCR

Total RNA was isolated by using commercial RNeasy® Mini Kit (Qiagen, 74104) and converted to the cDNA using high-capacity RNA-to-cDNA Kit (Applied Biosystems, 4368814). qPCR was performed on cells in the same time points for five assays: *Nestin* (Mm00450205_m1), *Map2* (Mm00485231_m1), *Gfap* (Mm01253033_m1), *Synapsin-1* (Mm00449772_m1) and *Neuroligin-1* (Mm02344305_m1). As housekeeping gene, *β-Actin* (ACTB MGB 4352933E) (TaqMan Gene Expression Assays) was used. All samples were made in triplicate with 1 μg cDNA in total volume 20 μL using Applied Biosystems 7500 Real-Time PCR System. Relative quantification was made using formula 2 − ΔCT.

### Imaging and image analysis

Confocal figures were captured by Olympus FV 3000 microscope using 60× objective and further analysed using IMARIS 9.9.1. (BITPLANE, An Oxford Instruments Co., Zurich, Switzerland) software - Neuroscience module. Quantification was performed on 5–8 full Z-stack images/staining/time point, using “surface”, “spot” and “filament” option as well as Pearson’s coefficient of colocalization. Each Z-stack contain over 80 DAPI positive nuclei. For the Filament analysis, following options were used: Filament Area, Filament Length, Filament No. Terminal Points, Sholl Intersections, Pearson’s coefficient of colocalization and synaptic activity. On the other words, Filament Area was defined as the sum of the areas of all processes per field of view, Filament length was defined as a length of all processes per field of view, Filament No. Terminal Points was defined as a number of terminal points per field of view, No. of Sholl Intersections was defined as the number of process intersections (branches) on concentric spheres (1.0 µm step), defining process spatial distribution as a function of distance from the beginning point. Pearson’s coefficient of colocalization is defined as corelation between two channels [39].

All data were presented as the mean ± standard error of the mean (Graphs represent means ± SEM). Statistical analyses were performed using PRISM (GraphPad Software Inc.) with Student’s *T* test when comparing single pair of samples while for comparisons of more than one sample, one-way ANOVA was carried out, followed by Bonferroni’s multiple comparisons test.

## Supplementary information

Supplementary document 1

Original data

Supplementary data

## Data Availability

Original data are available upon request.
