# Supplementary material for: Thy1-YFP: an effective tool for single cell tracing from neuronal progenitors to mature functionally active neurons
Source: Cell Death Discov. 2025 Jan 22;11:18. doi: 10.1038/s41420-025-02297-z (PMC11754755; doi:10.1038/s41420-025-02297-z)

β-Actin_membrane 1

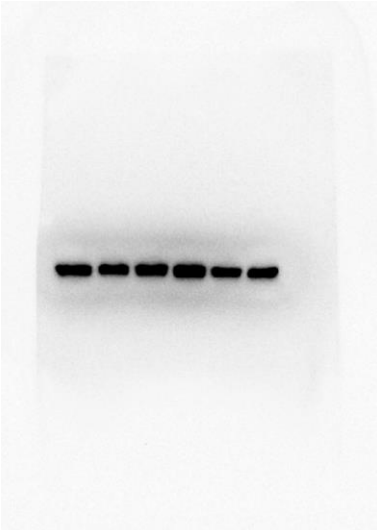

β-Actin_membrane 2

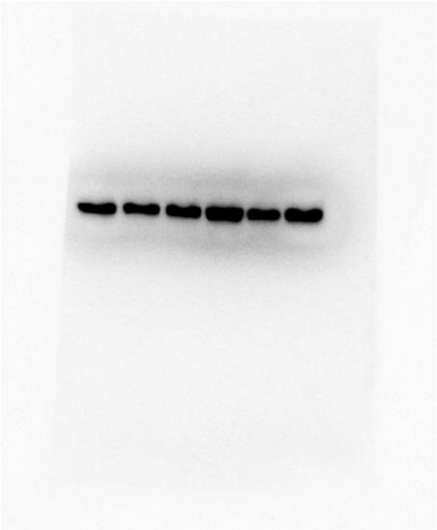

β-Actin_membrane 3

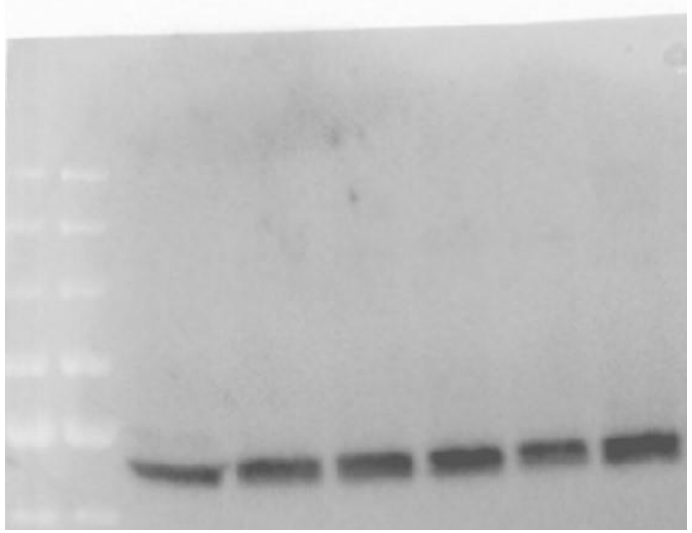

β-Actin_membrane 4

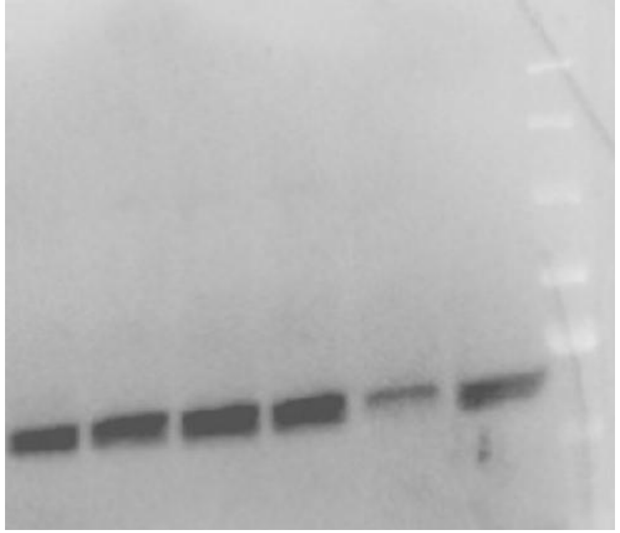

TUBB3_membrane 1

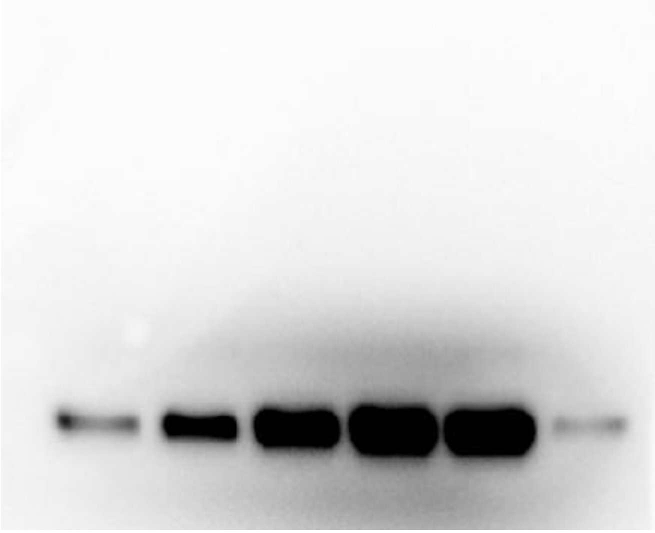

TUBB3_membrane 2

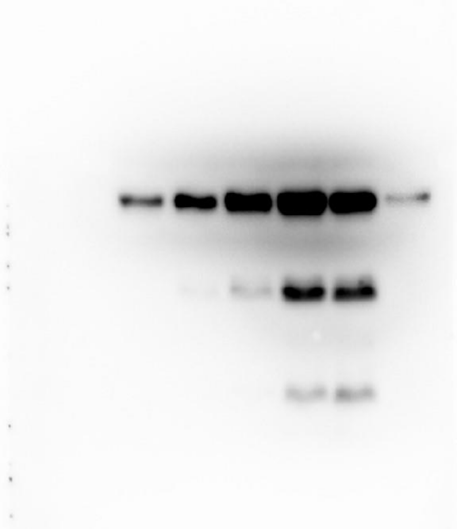

TUBB3_membrane 3

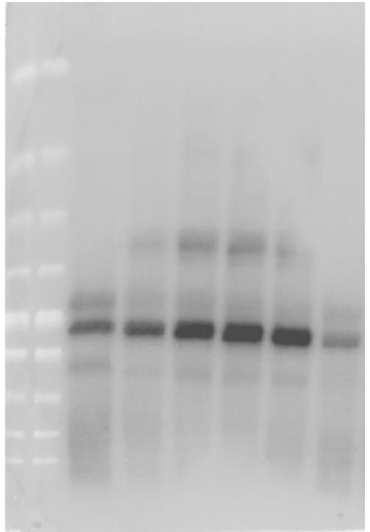

TUBB3_membrane 4

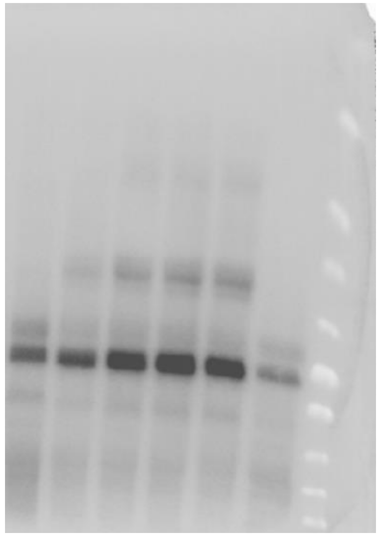

GFAP_membrane 1

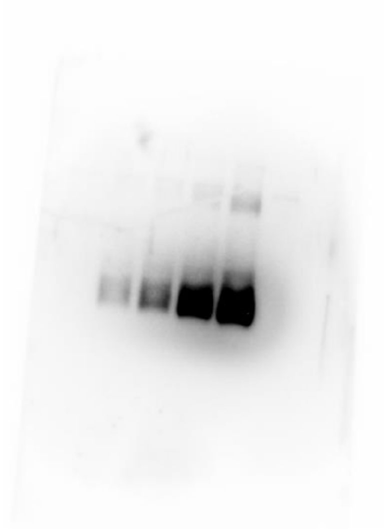

GFAP_membrane 2 and 3

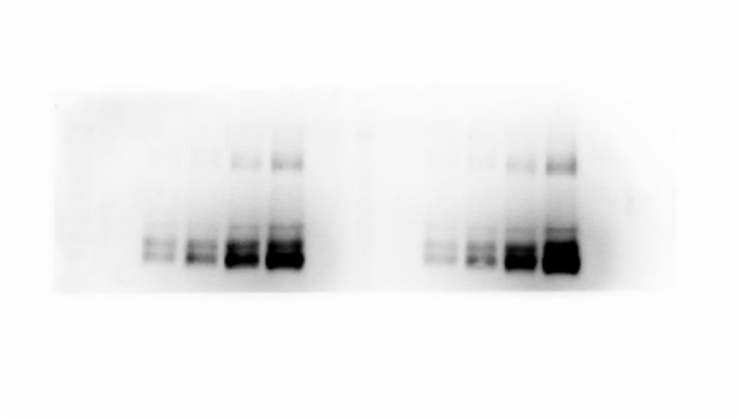

GFAP_membrane 4

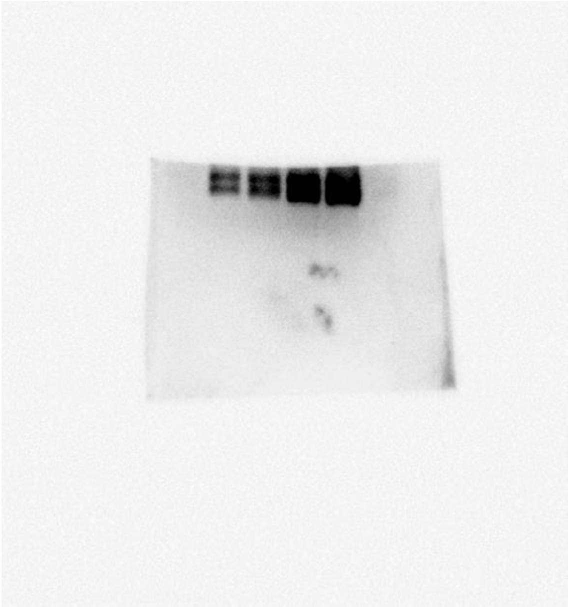

SOX2_membrane 1 and 2

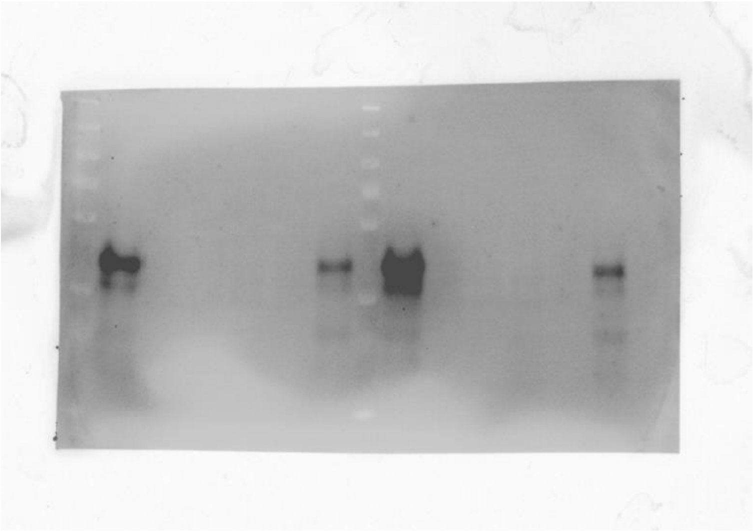

SOX2_membrane 3

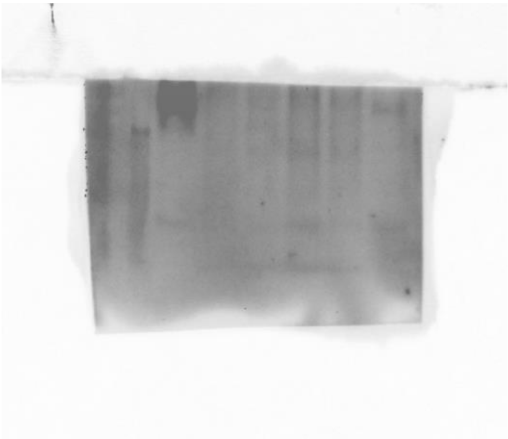

GFP_membrane 1 and 2

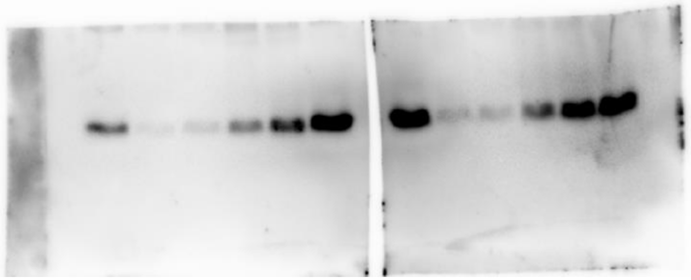

GFP_membrane 3

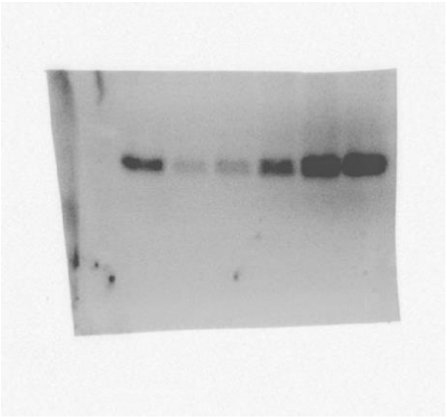

Supplement: Supplementary file 2 — Original data [file 41420_2025_2297_MOESM2_ESM.pdf]
