# Supplementary material for: Thy1-YFP: an effective tool for single cell tracing from neuronal progenitors to mature functionally active neurons
Source: Cell Death Discov. 2025 Jan 22;11:18. doi: 10.1038/s41420-025-02297-z (PMC11754755; doi:10.1038/s41420-025-02297-z)
Supplement: Supplementary file 3 — Supplementary data [file 41420_2025_2297_MOESM3_ESM.docx]

**Supplementary data**

**TITLE**: **Thy1-YFP: an effective tool for single cell tracing from neuronal progenitors to mature functionally active neurons**


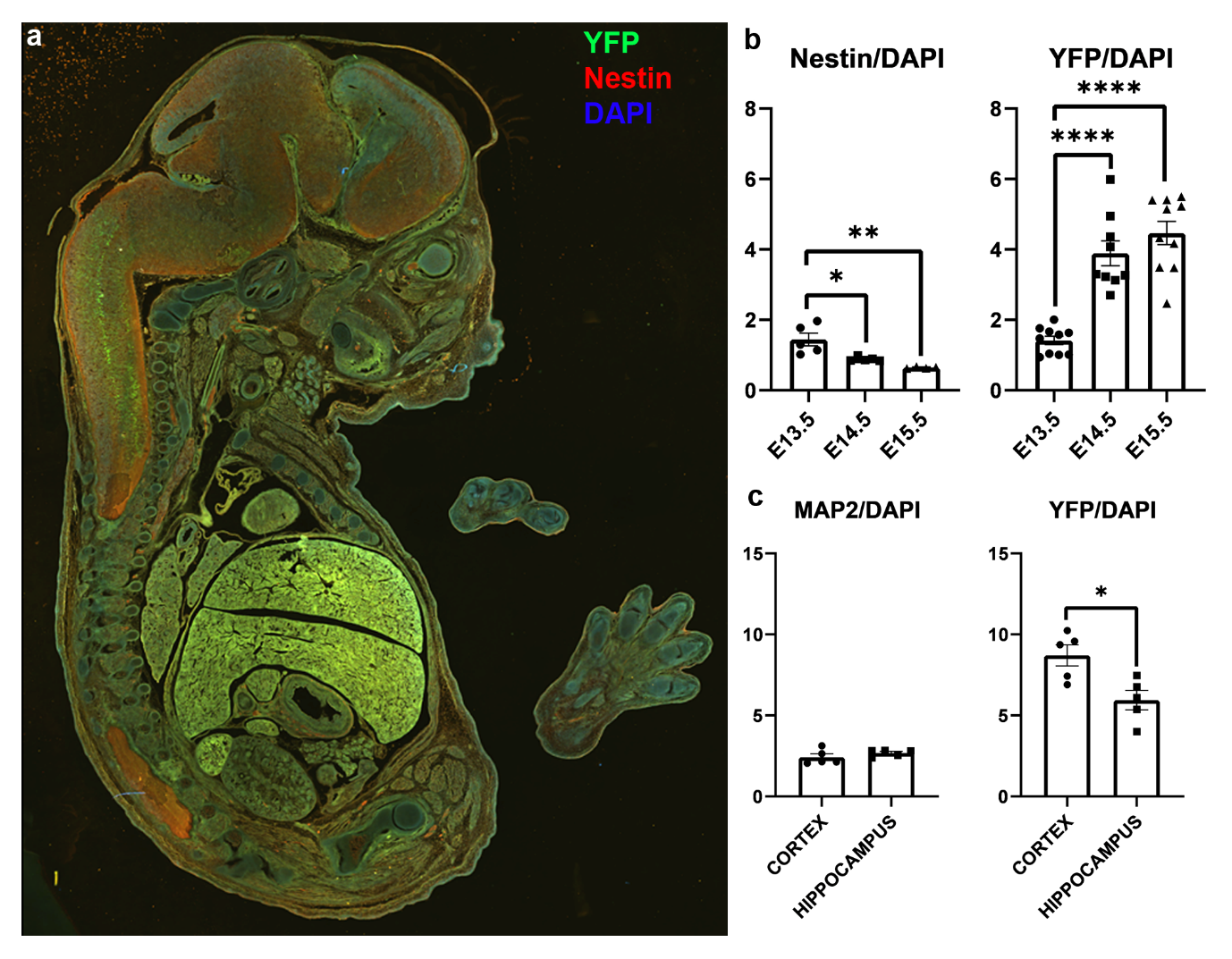


**Supplementary figure 1.** **Analysis of Nestin, MAP2 and YFP during embryonic development and at P0**. Representative figure of Thy1-YFP E14.5 stained with Nestin (red) (a). Quantification shows high expression of Nestin and YFP during E13.5, E14.5 and E15.5 per field of view, normalised with DAPI (b). Moreover, MAP2 expression was analysed at P0 and showing similar levels of expression in both the cortex and hippocampus (c). Significantly higher expression of YFP was obtained in the cortex compared to the hippocampus. Based on shown data, we used telencephalic wall of E14.5 and P0 for isolation and differentiation *in vitro*. *P* values are provided in the separate Supplementary document 1. Graphs represent means ± SEM.


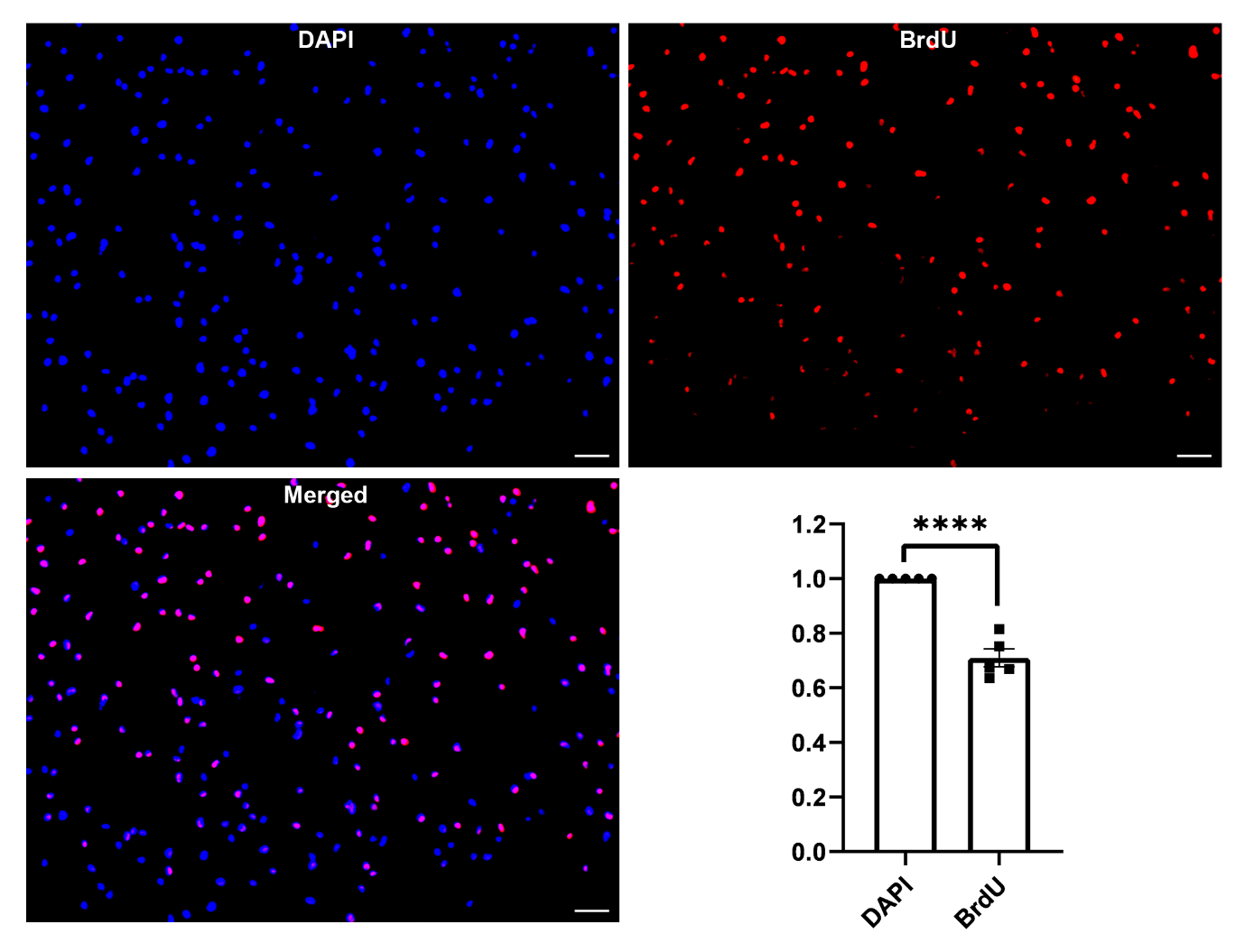


**Supplementary figure 2. Analysis of BrdU-labelled NSCs at DIV0**. After 8 hours of NSCs labelling with 10 µM BrdU solution, over 75% NSCs were BrdU positive, suggesting a high proliferation rate of NSCs under our experimental conditions. Scale bar 50 µm. *P* values are provided in the separate Supplementary document 1. Graphs represent means ± SEM.


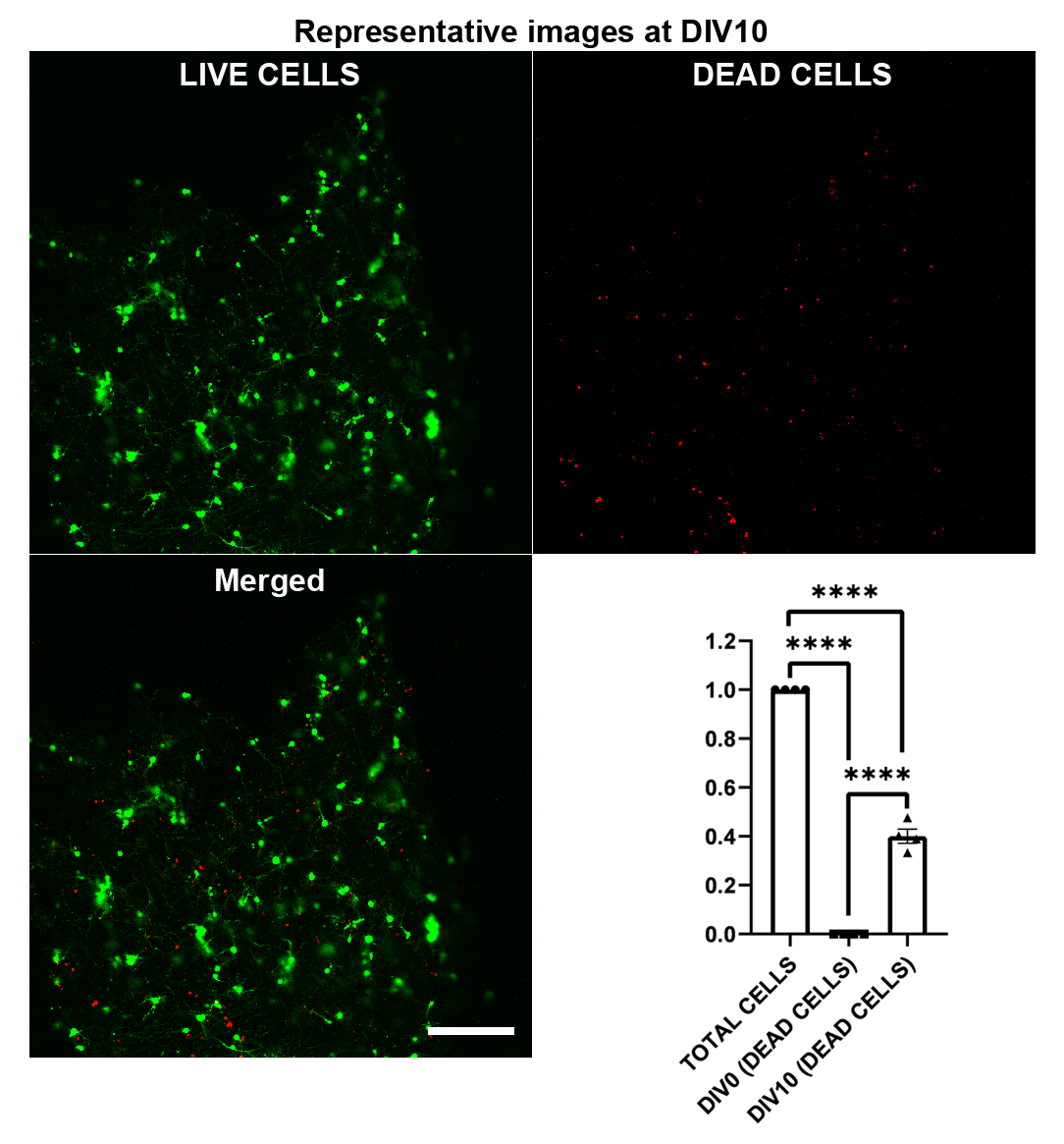


**Supplementary figure 3. Analysis of live/dead cells during differentiation *in vitro***. LIVE/DEAD staining was performed at DIV0 and DIV10. Live cells were labelled in green, while the dead cells were labelled in red (representative images of staining at DIV10 are shown). At DIV0 all cells were viable, while at DIV10 approximately 40% of the cells were dead. A significant difference was observed between the two time points. Scale bar 50 µm. *P* values are provided in the separate Supplementary document 1. Graphs represent means ± SEM.


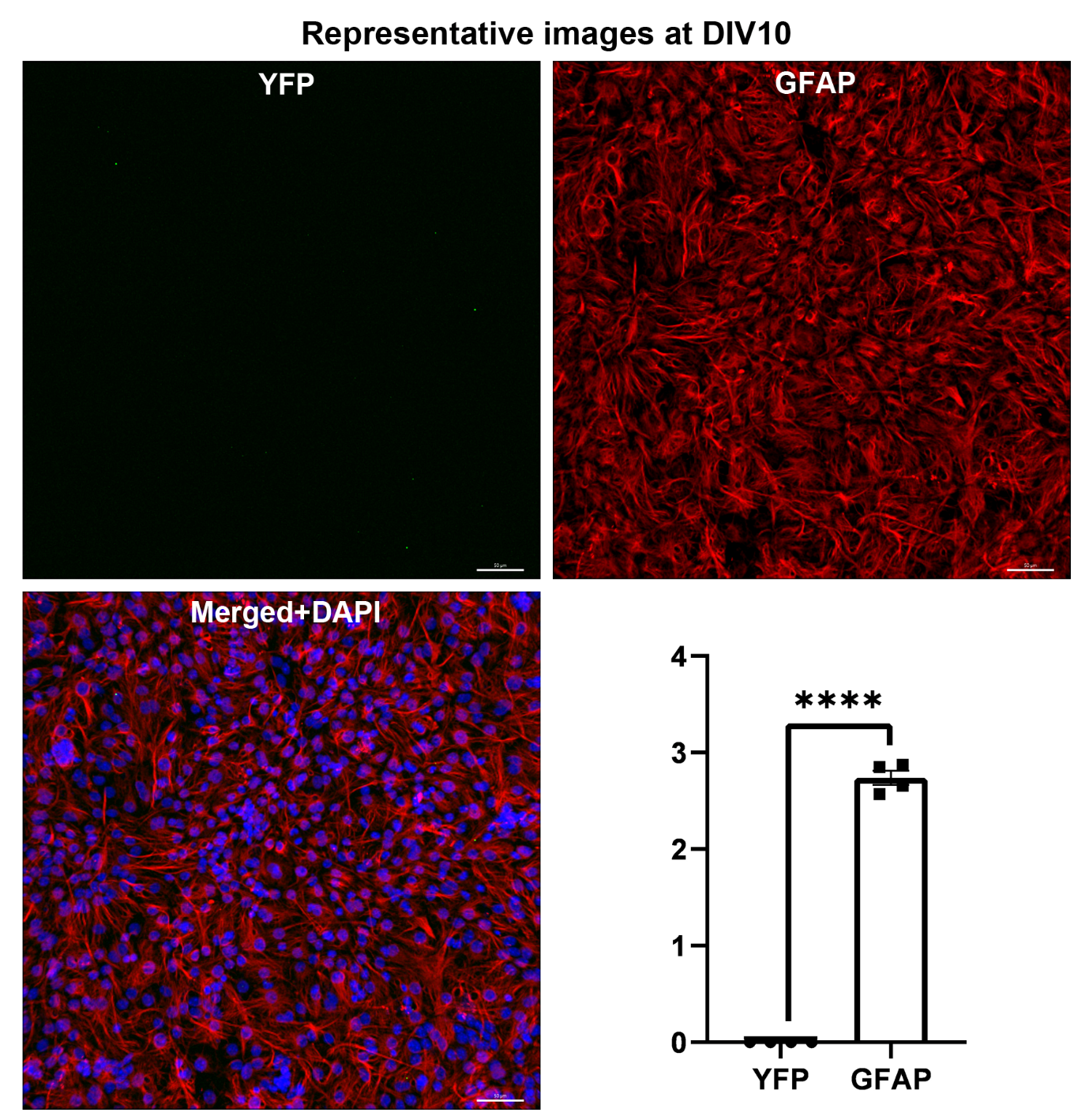


**Supplementary figure 4. Analysis of astrocytes differentiation *in vitro*.** NSCs were differentiated into astrocytes to show neuronal specificity of Thy1-YFP expression. Representative images of mature astrocytes show they were YFP negative. Scale bar 50 µm. *P* values were shown in the separate Supplementary document 1. Graphs represent means ± SEM.


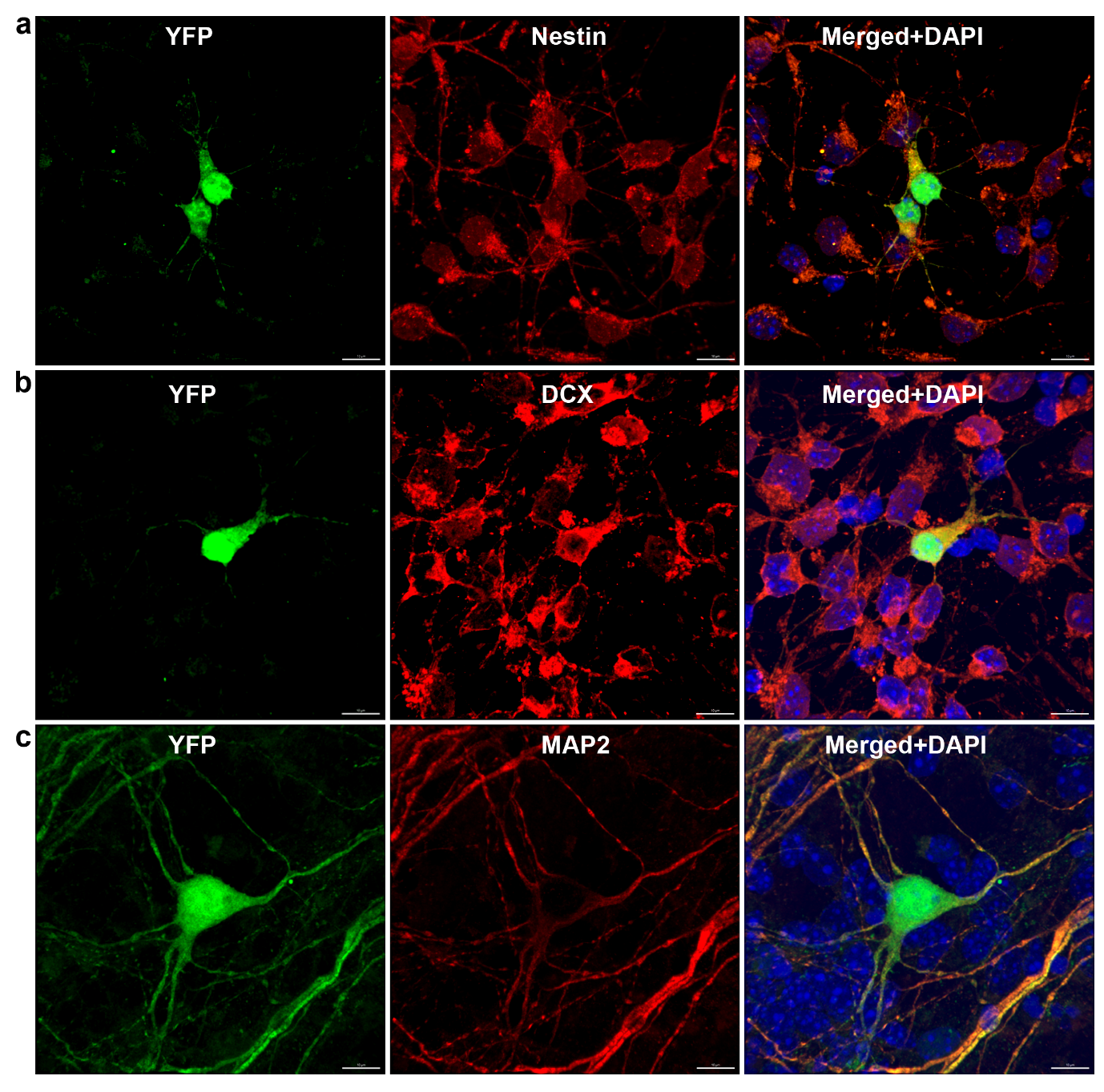


**Supplementary figure 5.** **Representative images of staining used for analysis shown in the main Figure 1**. NSCs at DIV1 labelled with Nestin (a). Immature neurons at DIV3 labelled with DCX (b) and Mature neurons at DIV10 labelled with MAP2 (c). Scale bar 10 µm.


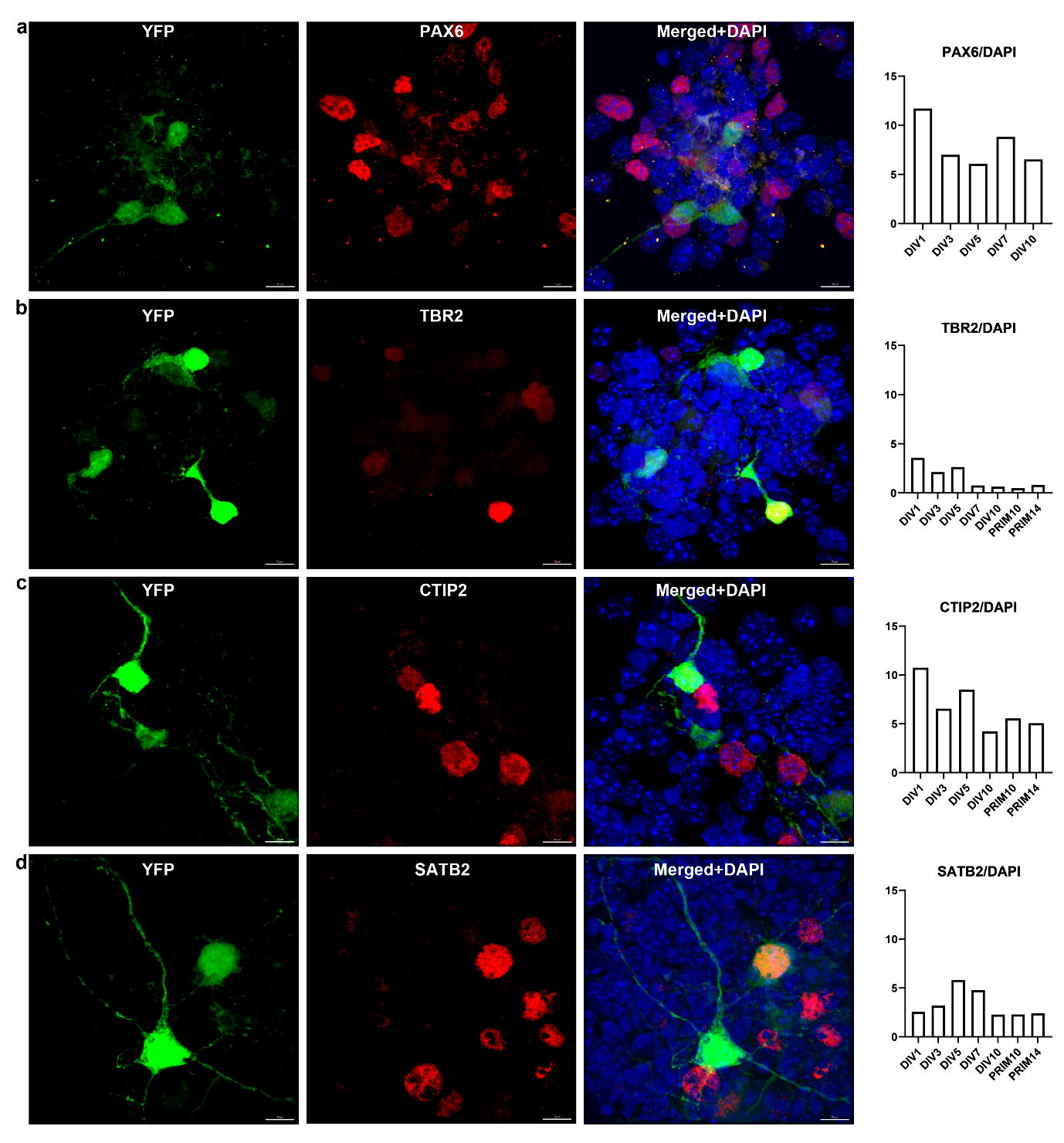


**Supplementary figure 6.** **Analysis of** **nuclear markers during differentiation *in vitro***. Representative images of immunostaining for PAX6 at DIV1 (a), TBR2 at DIV3 (b), CTIP2 at DIV5 (c) and SATB2 at DIV5 (d), along with their percentage of expression during differentiation *in vitro* (a-d). The expression of all markers remained consistent, with no significant differences observed between time points. Scale bar 10 µm.


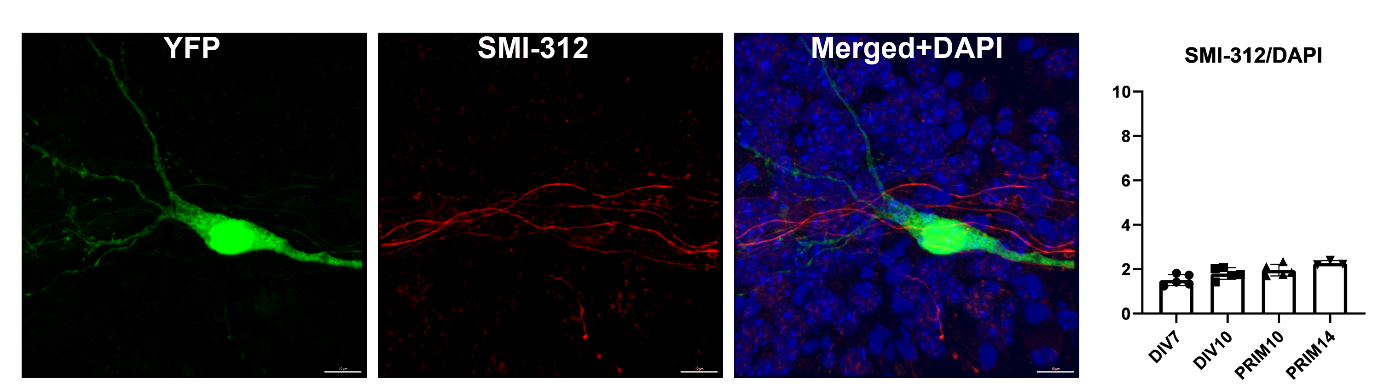


**Supplementary figure 7.** **Analysis of SMI-312 during differentiation *in vitro*.** Representative images of mature neurons/axons (SMI-312). SMI-312 positive axons were observed at DIV7 and DIV10, with no significant differences between time points. Scale bar 10 µm. Graphs represent means ± SEM.

**Supplementary Table 1**. List of primary antibodies used in this study.

| **Primary antibodies** | **Isotype** | **Dilution** | **Company** |
| --- | --- | --- | --- |
| β-Actin | Mouse monoclonal | 1:60000 | Sigma-Aldrich (A5441) |
| BrdU | Mouse monoclonal IgG1 | 1:200 | Cell Signaling (5292S) |
| CADM1 | Rabbit polyclonal | 1:200 | Sigma-Aldrich (ABT66) |
| Ctip2 | Rat IgG2a monoclonal | 1:200 | Abcam (ab18465) |
| Doublecortin | Goat IgG polyclonal | 1:1000 | Abcam (ab223435) |
| Gephyrin | Rabbit IgG monoclonal | 1:200 | Abcam (ab181382) |
| GFAP | Rat IgG2a monoclonal | 1:1000 | ThermoFisher SCIENTIFIC (13-0300) |
| GFAP | Chicken IgY polyclonal | 1:10000 | Abcam (ab4674) |
| GFP | Chicken IgY polyclonal | 1:5000 | Aves (1020) |
| MAP2 | Chicken IgY polyclonal | 1:1000 | Abcam (ab5392) |
| Nestin | Goat polyclonal | 1:200 | Santa Cruz Biotechnology (sc-21248) |
| Neurofilament Marker (pan axonal, cocktail, SMI-312) | Mouse IgG1 / IgM monoclonal | 1:1000 | Biolegend (837904) |
| NEUROLIGIN-1 | Rabbit polyclonal | 1:200 | Merck Millipore (AB15512) |
| PAX6 | Rabbit polyclonal | 1:200 | Merck Millipore (AB2237) |
| Piccolo | Mouse IgG2a monoclonal | 1:200 | Abcam (ab101654) |
| PSD95 | Rabbit IgG monoclonal | 1:400 | Cell Signaling (mAb #3450) |
| SATB2 | Rabbit IgG polyclonal | 1:200 | Abcam (ab34735) |
| SOX2 | Rabbit IgG monoclonal | 1:1000 | Cell Signaling (23064S) |
| SYNAPSIN-1 | Rabbit IgG monoclonal | 1:400 | Cell Signaling (#5297) |
| TBR2 | Rabbit IgG polyclonal | 1:200 | Abcam (ab23345) |
| TUBB3 | Rabbit IgG polyclonal | 1:5000 | Biolegend (802001) |

**Supplementary Table 2**. List of secondary antibodies used in this study.

| **Secondary antibodies** | **Dilution** | **Company** |
| --- | --- | --- |
| Donkey anti-Goat IgG (H+L) | 1:1000 | TermoFisher SCIENTIFIC (A11056) |
| Donkey anti-Mouse IgG (H+L) | 1:500 | TermoFisher SCIENTIFIC (A31571) |
| Goat anti-Chicken IgY (H+L) | 1:1000 | TermoFisher SCIENTIFIC (A11040) |
| Goat Anti-Chicken IgY H&L (HRP) | 1:10000 | Abcam (ab6877) |
| Goat anti-Rabbit IgG (H+L) | 1:500 | TermoFisher SCIENTIFIC (A21070) |
| Goat Anti-Rabbit IgG H&L (HRP) | 1:200000 | Abcam (ab6721) |
| Goat anti-Rat IgG (H+L) | 1:1000 | TermoFisher SCIENTIFIC (A21434) |
| Rabbit Anti-Mouse IgG H&L (HRP) | 1:200000 | Abcam (ab6728) |
